# Danhong injection for the treatment of early diabetic nephropathy: A protocol of systematic review and meta-analysis

**DOI:** 10.1097/MD.0000000000022716

**Published:** 2020-10-23

**Authors:** Caixia Huang, Cuiling Huang, Guomin Zhou

**Affiliations:** Department of Nephrology, Beijing Hospital of Integrated Traditional Chinese and Western Medicine, Beijing, China.

**Keywords:** Chinese medicine, danhong injection, early diabetic nephropathy, meta-analysis, systematic review

## Abstract

**Background:**

Diabetic nephropathy (DN) is the one that of the most common complications of diabetes mellitus (DM). Diabetic patients will experience a high mortality rate when DN progress to end-stage. So, it is extremely important to early treat DN. Although several interventions have been used to treat DN, a conclusive finding has not already been achieved. As one of the most common Chinese medicines, danhong injection (DHI) which has been shown to have various functions has also been prescribed to be as the alternative treatment option. However, no systematic review and meta-analysis has been conducted to objectively and comprehensively investigate its effectiveness and safety. Thus, we designed the current systematic review and meta-analysis to answer whether DHI can be preferably used to timely treat DN.

**Methods:**

We will perform a systematic search to capture any potentially eligible studies in several electronic databases including PubMed, Cochrane library, Embase, China National Knowledgement Infrastructure (CNKI), Wanfang database, and Chinese sci-tech periodical full-text database (VIP) from their inception to August 31, 2020. We will assign 2 independent reviewers to select eligible studies, and assess the quality of included studies with Cochrane risk of bias assessment tool. We will perform all statistical analyses using RevMan 5.3 software.

**Ethics and dissemination:**

We will submit our findings to be taken into consideration for publication in a peer-reviewed academic journal. Meanwhile, we will also communicate our findings in important conferences.

**Protocol registry:**

The protocol of this systematic review and meta-analysis has been registered at the International Plateform of Registered Systematic Review and Meta-Analysis Protocols (INPLASY) platform (https://inplasy.com/inplasy-2020-9-0005/, registry number: INPLASY202090005) and this protocol was funded through a protocol registry.

## 1 Introduction

Diabetes mellitus (DM) remains one of the major health issues, and the estimated number of patients diagnosed with DM will exceed 500 million in 2030 around the world.^[1]^ Patients with a long history of DM has been proved to be associated with associated with a great deal of serious complications such as diabetic retinopathy and nephropathy.^[2,3]^ As one of the most common complications of DM, diabetic nephropathy (DN) is associated with the increased mortality in diabetic patients with end-stage kidney disease.^[4]^ Moreover, the number of diabetic patients with DN account for 20 to 40% of the number of DM patients as the spreading of epidemic of DM.^[5]^ Therefore, it is imperative to effectively treat it when patients are identified at the early stage of DN in order to greatly reduce the negative impact of DN on prognosis and health-related quality of life.^[6–9]^

To date, several treatment options such as renin-angiotensin system blockers^[10]^ and omega-3 fatty acids^[11]^ have been developed to reduce the progression of kidney damage and then reduce the occurrence of complications resulted from DN.^[12]^ However, these regimes have not potential of preventing or reversing DN,^[13]^ and some regimes have been reported to be associated with increased incidence of adverse events and serious complications.^[14]^ Meanwhile, some of treatment regimes have also been suggested to be contraindicated for patients with severe renal impairment and have a set of serious side effects.^[15]^ As a result, researchers and practitioners have changed their attention from western medicine alone to Chinese medicine alone or integrated regime of western and Traditional mediciness.^[6,14,16]^

As one of the common use of Chinese medicine, danhong injection (DHI), which is manufactured with aqueous extracts of Radix Salviae Miltiorrhizae and Flos Carthami tinctorii,^[2]^ has been prescribed to treat a variety of conditions in mainland China for many years^[17–19]^ because it has been demonstrated to have the following functions: vasodilation, decreasing vascular resistance and blood viscosity, recovering neurological function, reducing inflammatory responses, activating platelet inhibition, and improving blood pressure.^[19]^ It is important that DHI has also been used to treat early diabetic nephropathy, however no consensus about its efficacy and safety has been achieved.^[20,21]^ Therefore, we designed this systematic review and meta-analysis to objectively investigate the clinical performance of DHI for the treatment of early diabetic nephropath.

## 2 Methods

### 2.1 Study registration

We performed the present protocol of systematic review and meta-analysis of randomized controlled trials (RCTs) in accordance with the methodological framework developed by the Cochrane Collaboration.^[22]^ Moreover, we also followed the recommendations reported in preferred reporting items for systematic review and meta-analysis protocols (PRISMA-P) 2015 statement.^[23]^ Moreover, we also registered our protocol on the International Plateform of Registered Systematic Review and Meta-Analysis Protocols (INPLASY) platform (https://inplasy.com/inplasy-2020-9-0005/, registry number: INPLASY202090005).^[24]^ And thus, our study protocol was funded through a protocol registry. We did not applied ethical approval and patients informed consent because all statistical analyses in our systematic review and meta-analysis would be performed based on published data.

### 2.2 Selection criteria

According to our aims, we designed the following inclusion criteria: (a) all randomized controlled trials (RCTs) which were performed to investigate the comparative efficacy and safety of DHI alone versus conventional regime such as diet restriction or western medicine in diabetic patients with early DN will be considered for eligibility; (b) adult diabetic patients regardless sex are diagnosed as early DN in accordance with definitive diagnosis standards which must be introduced in details; (c) the information of at least one of effectiveness and safety can be accessed; (d) only studies published in English and Chinese language will be eligible for our inclusion criteria.

We will exclude a study if it cover at least one of the following criteria: (a) essential information for the final analysis is not available; (b) duplicate records with less information; (c) ineligible study design such as narrative review, commentary, case report or series, animal study.

### 2.3 Definition of outcomes

In our systematic review and meta-analysis, we will design effective rate as the primary outcome, which will be determined in accordance with the definitive Chinese traditional medicine effectiveness evaluation criteria. According to evaluation criteria, the effective rate is the ratio of the number of patients who are identified to have healing, significant effect, and effect divided by the number of all patients who are assigned to a certain intervention group.^[14]^ We will define 24-hour urine protein quantitation, urinary albumin excretion rate, fasting blood glucose, and glycosylated hemoglobin as the secondary outcomes, which are all quantitatively identified by laboratory test. Moreover, we will calculate the adverse events (AEs) which are associated with interventions as the secondary outcomes, which are all recorded by individual study.

### 2.4 Identification of studies

**Figure 1:**
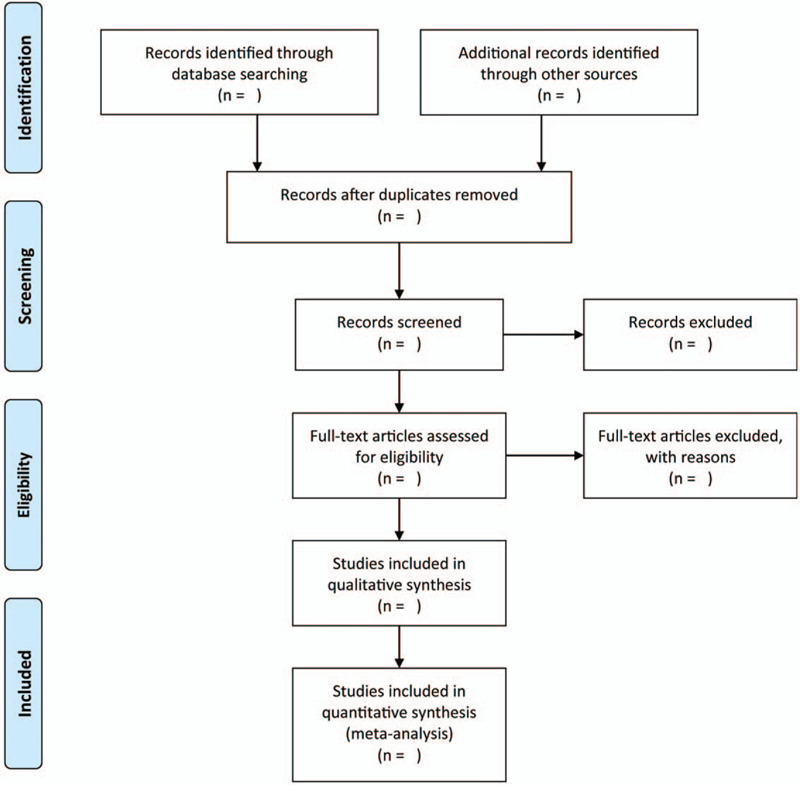
The flow-chart of identifying and selecting eligible studies.

For the purpose of capturing all potentially eligible studies, we will assign 2 independent reviewers to perform a systematic search in several electronic databases including PubMed, Cochrane library, Embase, China National Knowledgement Infrastructure (CNKI), Wanfang database, and Chinese sci-tech periodical full text database (VIP). The search time will be limited from their inception to August 31, 2020. Moreover, we will update search results weekly in order to capture any additional studies. We will use the following core words to construct the sensitive search strategy: “danhong injection”, “early diabetic nephropathy”, and “random”. The combination of full text words and medical subject heading (MeSH) will be used in order to guide developing a search strategy. As an example, we documented the PubMed search query in Supplementary Table 1. We will also check reference lists of all included studies and reviews which were carried out to summarize the evidence of DHI for the treatment of early diabetic nephropathy in order to capture any potentially eligible studies. We will resolve disagreements about identification of studies through consulting with third senior reviewer. The progress of identifying and selecting eligible studies will be depicted in Figure 1.

### 2.5 Data extraction

We will assign 2 reviewers to independently extract the essential information with standard data extraction sheet. We will extract the following for the final analysis: the leading authors name, country of the leading author, year of including a formal volume and issue, sample size, gender ratio, age, duration of DM, level of blood glucose at the baseline, details of interventions, outcomes of interesting, and details of risk of bias. We will resolve disagreements about data extraction through consulting with third senior reviewer. All basic characteristics of included eligible studies will be summarized in Table 1.

**Table 1:**
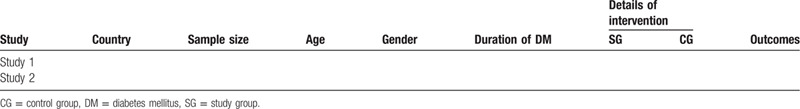
Basic characteristics of all included studies.

### 2.6 Assessment of risk of bias

The overall quality of all included studies is associated with the reliability and robustness of pooled results. Therefore, we will critically evaluate the quality of included study with Cochrane risk of bias assessment tool.^[25]^ Each included study will be assessed from the following 6 domains including randomization sequence generation, allocation concealment, blinding of participants, blinding of study personnel, blinding of outcome assessors, incomplete outcome data, selective reporting and other bias.^[22,25]^ Accoridng to the actual information of each study in terms of risk of bias, individual study will be labeled with “low risk of bias”, “unclear risk of bias”, and “high risk of bias”.^[25]^ The overall level of all included studies will be determined according to the results of assessing the risk of bias of individual study.^[22]^

### 2.7 Statistical analysis

In our systematic review and meta-analysis, we will calculate the relative risk (RR) with 95% confidence intervals (CIs) to express dichotomous data, and the mean difference (MD) with 95% CIs to express continuous data. Before performing statistical analysis, we will firstly use the Cochrane Q test to qualitatively assess the heterogeneity across included studies,^[26]^ and then we will use *I*^2^ statistic to quantitatively estimate heterogeneity.^[27]^ We will consider included studies for individual outcome as heterogeneity if *I*^2^ > 50% and *P* < .10.^[22]^ In contrast, studies will be considered as homogeneous when a *I*^2^ ≤ 50% and a *P* ≥ .10 was estimated.^[22]^ We will perform all statistical analyses based on a random-effect model because no homogeneous studies will be found in the real world. All statistical analyses will be conducted with the RevMan 5.3 (Copenhagen, Denmark: the Nordic Cochrane Center, the Cochrane Collaboration, 2013).

### 2.8 Subgroup and sensitivity analyses

In order to eliminate the impact of critical confounding factors on all statistical analyses, we will perform several subgroup analyses according to the duration of DM and intervention regimes in control group. We will also check the robustness of pooled results through excluding eligible studies with high risk of bias.^[22]^

### 2.9 Publication bias

We will draw funnel plot to qualitatively inspect the publication bias of individual outcome when accumulated number of included studies were more than 10.^[28,29]^ We will make a decision that absence of publication bias when an asymmetric funnel plot was generated.^[29]^

## 3 Discussion

### 3.1 Rational basis of performing meta-analysis

With the increasing of the total number of patients with diagnosed as DM, the proportion of patients with DN is also increasing.^[3]^ Considering the high incidence of serious consequences when DN was progressed to end-stage, a great deal of interventions have been developed in order to effectively treat it when DN was identified at the early stage.^[10,11,15]^ However, most of the interventions have not potential of preventing or reversing DN, so no optimal treatment option has been reported presently.^[12]^ Although researchers and practitioners have also changed attentions from western medicine to traditional Chinese medicine, and a series of clinical studies and meta-analyses have also been performed to investigate the efficacy and safety of some Chinese medicines for the treatment of early DN,^[6,14,15]^ it remains to be answered about optimal regime. Thus, it is important to explore the effectiveness and safety of other Chinese medicine.

DHI is one of the most common Chinese medicine in mainland China, and it has been widely used in clinical practices due to several functions such as promoting blood circulation, removing blood stasis and dredging meridians, reducing blood viscosity, and scavenging free radicals.^[30–33]^ Considering these functions, DHI has also been used to early treat DN in clinical practice.^[6,20]^ To date, several clinical studies have been reported, however no systematic review and meta-analysis to comprehensively investigate the effectiveness and safety through calculating all results from various individual studies.

### 3.2 The importance of main findings

We designed the present systematic review and meta-analysis to firstly investigate the effectiveness and safety of DHI for the prevention and treatment of early DN. When we completed the current systematic review and meta-analysis, more reliable and robust findings will be generated, which will provide more information for developing intervention regimes for the prevention and treatment of early DN.

### 3.3 Ethics and dissemination

We do not need ethics approval and informed consent in the current systematic review and meta-analysis because we will perform all statistical analyses based on published data. After completed this systematic review and meta-analysis, we will submit it to be considered for publication in a peer-reviewed scholarly journal. Moreover, we will also submit our findings to gain more communications in some important conferences.

## Acknowledgments

We would like to express our warm appreciation for the International Plateform of Registered Systematic Review and Meta-Analysis Protocols (INPLASY) platform for registry for our systematic review and meta-analysis.

## Author contributions

CXH conceived and designed this protocol. CXH and CLH reviewed scoping searches and contributed to the methodologic development of the protocol. CXH drafted the initial manuscript and remaining 2 authors (CLH, GMZ) revised it. All the authors have given approval of publishing. GMZ is the review guarantor.

**Conceptualization:** Caixia Huang.

**Data curation:** Caixia Huang.

**Formal analysis:** Caixia Huang, Cuiling Huang.

**Investigation:** Caixia Huang, Cuiling Huang.

**Methodology:** Caixia Huang, Cuiling Huang.

**Software:** Caixia Huang, Cuiling Huang.

**Supervision:** Caixia Huang, Guomin Zhou.

**Validation:** Caixia Huang, Guomin Zhou.

**Visualization:** Caixia Huang, Guomin Zhou.

**Writing – original draft:** Caixia Huang.

**Writing – review & editing:** Caixia Huang, Guomin Zhou.

## Supplementary Material

Supplemental Digital Content
