# Supplementary material for: Danhong injection for the treatment of early diabetic nephropathy: A protocol of systematic review and meta-analysis
Source: Medicine (Baltimore). 2020 Oct 23;99(43):e22716. doi: 10.1097/MD.0000000000022716 (PMC7581143; doi:10.1097/MD.0000000000022716)
Supplement: Supplemental Digital Content [file medi-99-e22716-s001.docx]

| **PubMed search query** | |
| --- | --- |
| Search number | Query |
| 6 | ((("Diabetic Nephropathies"[Mesh]) OR (((((((((((Diabetic Nephropathies[Title/Abstract]) OR (Diabetic Nephropathy[Title/Abstract])) OR (Diabetic Kidney Disease[Title/Abstract])) OR (Diabetic Kidney Diseases[Title/Abstract])) OR (Diabetic Glomerulosclerosis[Title/Abstract])) OR (Kimmelstiel-Wilson Syndrome[Title/Abstract])) OR (Kimmelstiel Wilson Syndrome[Title/Abstract])) OR (Kimmelstiel-Wilson Disease[Title/Abstract])) OR (Kimmelstiel Wilson Disease[Title/Abstract])) OR (Nodular Glomerulosclerosis[Title/Abstract])) OR (Intracapillary Glomerulosclerosis[Title/Abstract]))) AND (((Danhong injection[Title/Abstract]) OR (Danhong injections[Title/Abstract])) OR (DHI[Title/Abstract]))) AND (random*) |
| 5 | random* |
| 4 | ((Danhong injection[Title/Abstract]) OR (Danhong injections[Title/Abstract])) OR (DHI[Title/Abstract]) |
| 3 | ("Diabetic Nephropathies"[Mesh]) OR (((((((((((Diabetic Nephropathies[Title/Abstract]) OR (Diabetic Nephropathy[Title/Abstract])) OR (Diabetic Kidney Disease[Title/Abstract])) OR (Diabetic Kidney Diseases[Title/Abstract])) OR (Diabetic Glomerulosclerosis[Title/Abstract])) OR (Kimmelstiel-Wilson Syndrome[Title/Abstract])) OR (Kimmelstiel Wilson Syndrome[Title/Abstract])) OR (Kimmelstiel-Wilson Disease[Title/Abstract])) OR (Kimmelstiel Wilson Disease[Title/Abstract])) OR (Nodular Glomerulosclerosis[Title/Abstract])) OR (Intracapillary Glomerulosclerosis[Title/Abstract])) |
| 2 | ((((((((((Diabetic Nephropathies[Title/Abstract]) OR (Diabetic Nephropathy[Title/Abstract])) OR (Diabetic Kidney Disease[Title/Abstract])) OR (Diabetic Kidney Diseases[Title/Abstract])) OR (Diabetic Glomerulosclerosis[Title/Abstract])) OR (Kimmelstiel-Wilson Syndrome[Title/Abstract])) OR (Kimmelstiel Wilson Syndrome[Title/Abstract])) OR (Kimmelstiel-Wilson Disease[Title/Abstract])) OR (Kimmelstiel Wilson Disease[Title/Abstract])) OR (Nodular Glomerulosclerosis[Title/Abstract])) OR (Intracapillary Glomerulosclerosis[Title/Abstract]) |
| 1 | "Diabetic Nephropathies"[Mesh] |
